# Artificial Intelligence Models for the Automation of Standard Diagnostics in Sleep Medicine—A Systematic Review

**DOI:** 10.3390/bioengineering11030206

**Published:** 2024-02-22

**Authors:** Maha Alattar, Alok Govind, Shraddha Mainali

**Affiliations:** 1 Division of Adult Neurology, Sleep Medicine, Vascular Neurology, Department of Neurology, Virginia Commonwealth University, Richmond, VA 23284, USA; 2 Department of Neurology, National Institute of Mental Health and Neurosciences, Bangalore 560029, India; 3 Division of Vascular Neurology and Neurocritical Care, Department of Neurology, Virginia Commonwealth University, Richmond, VA 23284, USA

**Keywords:** artificial intelligence, sleep medicine, sleep disorders, deep learning, sleep and AI

## Abstract

Sleep disorders, prevalent in the general population, present significant health challenges. The current diagnostic approach, based on a manual analysis of overnight polysomnograms (PSGs), is costly and time-consuming. Artificial intelligence has emerged as a promising tool in this context, offering a more accessible and personalized approach to diagnosis, particularly beneficial for under-served populations. This is a systematic review of AI-based models for sleep disorder diagnostics that were trained, validated, and tested on diverse clinical datasets. An extensive search of PubMed and IEEE databases yielded 2114 articles, but only 18 met our stringent selection criteria, underscoring the scarcity of thoroughly validated AI models in sleep medicine. The findings emphasize the necessity of a rigorous validation of AI models on multimodal clinical data, a step crucial for their integration into clinical practice. This would be in line with the American Academy of Sleep Medicine’s support of AI research.

## 1. Introduction

Sleep, an essential biological requirement fundamental to human health and wellbeing, is a natural physiological state that is also vulnerable to various diseases and disorders. A significant portion of the general population experiences sleep disturbances, leading to a diminished quality of life, increased health risks, and higher healthcare expenses [1]. The National Sleep Foundation’s 2020 survey found that nearly half of Americans suffer from daytime sleepiness [2]. Additionally, between 35 and 50% of adults annually experience insomnia symptoms [3]. Sleep disorders, as classified by the International Classification of Sleep Disorders, include a range of conditions such as insomnia, sleep-related breathing disorders, hypersomnolence, circadian rhythm disruptions, parasomnias, and movement disorders during sleep [4].

**Figure 1:**
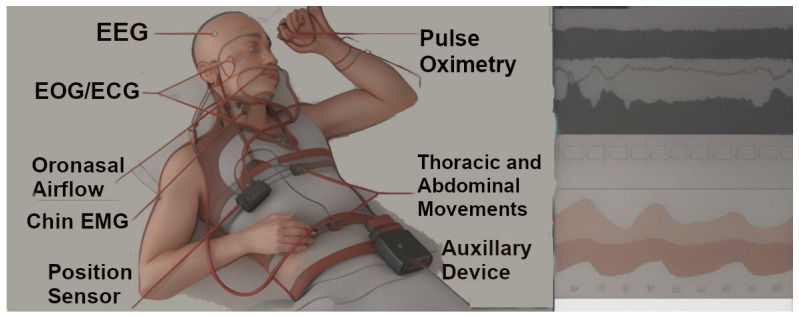
Image depicts a polysomnography study setup with multimodal monitoring tools: EEG (Electroencephalogram)—Electrodes attached to the head for monitoring brain wave activity; EOG/ECG (Electrooculogram/Electrocardiogram)—Equipment monitoring eye movements and heart activity; Oronasal Airflow Monitor—Device positioned near the nose and mouth to measure breathing; Chin EMG (Electromyogram)—Sensors attached to the chin to detect muscle activity; Position Sensor—A sensor placed on the body to detectsleep positions; Pulse Oximetry—A small device attached to a finger to measure blood oxygen levels and heart rate; Thoracic and Abdominal Movement Sensors—Sensors placed on the chest and abdomen to monitor respiratory effort and movement; Auxiliary Device—central component of the polysomnography system and it contains specialized amplifiers, filters, and computer chips that translate the signals collected from various bioelectrical potentials such as sensors and electrodes that is attached to the patient’s body into records that can be analyzed and visualized.

The field of sleep medicine is rich in clinical data, with nearly one million polysomnographic tests (Figure 1) conducted each year in the U.S. for analysis [5]. These disorders present unique challenges to patients and healthcare providers, particularly due to the altered state of awareness during sleep. Advancements in technology, especially in artificial intelligence (AI), present an opportunity for sleep medicine to transform. AI can efficiently process and analyze vast amounts of digital health data from both inpatient and outpatient sources, enabling the development of predictive diagnostic and treatment models. AI tools are adept at cleaning data, classifying diseases, and identifying specific disease patterns, tasks beyond the scope of human biological intelligence. Given that each patient generates over 80 megabytes of clinical data annually, a number that is only increasing, manually reviewing each patient’s data within the limited time of clinical sessions is becoming increasingly challenging [6]. This growth in data underlines the need for advanced technological solutions in diagnosing and managing sleep disorders.

In 1936, Alan Turing constructed the first computer model called the Turing machine, which laid the foundation for computation and artificial intelligence [7]. It was not until 1956 that computer scientists coined the term “artificial intelligence” during a workshop at Dartmouth College, marking the official beginning of the modern AI field [8]. In subsequent years, computing technology made its way into the medical realm, ushering in a new era of modern medicine. In 2007, IBM created WATSON (version 18.4), a computer software program used for decision management in lung cancer [9]. A decade later, in 2017, the first AI-based sleep device, EnsoSleep (EnsoData, Madison, WI, USA), received FDA approval for use in sleep medicine [10]. This software program automatically scores sleep stages and detects respiratory events, movements, and arousals from polysomnograms. It also aligns with the current guidelines of the American Academy of Sleep Medicine (AASM), the leading professional society for the promotion of sleep health. In 2019, the FDA approved WatchPat (Itamar Medical Inc., Atlanta, GA, USA), a wrist-worn device with substernal sensors, as a home sleep apnea diagnostic test [10]. It provides a non-invasive and accessible means for diagnosing sleep apnea.

Despite the widespread prevalence of sleep disorders, many patients go undiagnosed or do not adhere to treatment plans. This problem is partly due to the limited availability and high costs of sleep medicine clinics and laboratories, which are predominantly located in major urban areas. Obstructive sleep apnea (OSA), increasingly prevalent in the United States party due to the obesity epidemic, affects a substantial segment of the population but remains largely undiagnosed [11,12]. OSA, if not treated, heightens the risk of serious health issues such as hypertension, stroke, depression, and increased mortality [11]. Similarly, untreated insomnia is moderately linked to acute myocardial risk and a higher likelihood of stroke [13,14], and individuals with insomnia are twice as likely to develop depression compared to those without sleep issues [15]. REM sleep behavior disorder (RBD), marked by aggressive dreams and physical actions like kicking and vocal outbursts, is essential for the early detection of neurological disorders such as Parkinson’s disease due to its association with alpha-synucleinopathies. Central disorders of hypersomnolence, such as narcolepsy, cause significant daytime dysfunction. Sleep-related movement disorders, like periodic limb movements of sleep (PLMS), disrupt sleep continuity, leading to non-restorative sleep. Furthermore, sleep disorders contribute to elevated healthcare utilization and costs in the United States, with an estimated overall healthcare expenditure of approximately $94.9 billion [1].

In spite of the growing number of accredited sleep centers, there remains a significant gap in healthcare access to sleep medicine in the United States, where the ratio of people to sleep specialists is over 43,000 to 1 [16]. For instance, the effective management of OSA with Continuous Positive Airway Pressure (CPAP) therapy requires regular follow-ups at clinics, yet the capacity of specialized sleep medicine clinics to meet increasing demand is limited. Replicating the specialized services of these clinics in general settings, like primary care, is not feasible. Additionally, current treatments lack customization; they often fail to fully consider the complexity, heterogeneity, and genetic factors of each sleep disorder. AI-based tools offer a solution to these challenges by enhancing the capabilities of sleep clinics to improve access and treatment adherence. AI can identify complex patterns through predictive models that may be missed by humans and traditional statistical methods. It also reduces the workload of labor-intensive tasks, allowing healthcare professionals to focus more on direct patient care. Integrating AI into sleep medicine can optimize resource use, improve care access, and cut costs.

Bioengineers and clinicians are particularly interested in the automated processing of vast amounts of electrophysiologic data from sleep studies, achieved using Machine Learning (ML) models. These ML models, a subset of AI, use algorithms to identify patterns in data, aiding in classification and prediction tasks. With advances in the field of AI, the application of ML for the automated analysis of sleep studies has gained popularity. Additionally, the availability of public sleep datasets, comprising thousands of recordings from sleep labs and research studies, has facilitated the development of ML models by providing ample data for training. A multitude of ML models that have been designed to detect and label sleep-related events have been reported in recent years. The aim of this article is to select and review ML models that were developed using the rigorous methodology described below to automate standard diagnostic techniques that are used in the clinical practice of sleep medicine. Technical terms related to Machine Learning with respective examples are highlighted in Table 1.

### 1.1. Training, Testing, and Validation

In Machine Learning (ML), the development of a model begins with the training phase, where the model is exposed to a specific dataset, known as the training dataset. During this phase, the model learns to identify and interpret patterns within the data. Following this, the model undergoes a validation phase, where its accuracy is assessed using a separate sample of data, termed the validation dataset. This phase is crucial for fine-tuning the model, ensuring that it not only adheres closely to the training data but also generalizes effectively to new, unseen data. After successful training and validation, the model’s performance is evaluated on a different dataset, known as the “test” dataset. The results of this evaluation on the test dataset are critically reported.

A prevalent practice in ML is cross-validation, which involves using portions of the training data for validation purposes. Although cross-validation helps to mitigate overfitting (Table 1), using an entirely separate dataset for validation, one that the model has not encountered during its training phase, ensures a more accurate assessment of the model’s capability to generalize. Such a stringent approach is warranted in scenarios where precise and reliable model predictions are crucial, healthcare being a relevant example [17,18]. In this review, our focus is on studies that have adhered to this rigorous standard as we report the development of ML models using validation datasets that are distinct and separate from their training sets, thereby ensuring a higher degree of reliability and validity in their findings.

### 1.2. Deep Learning and Neural Networks

**Figure 2:**
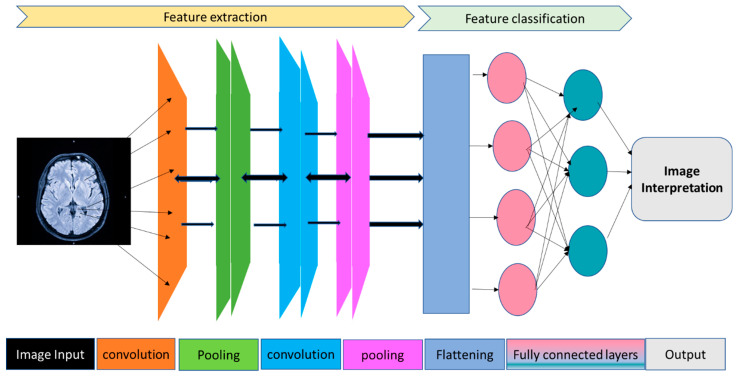
This figure illustrates a Convolutional Neural Network (CNN) designed for classifying neuroimaging data. The structure includes the following: Input Layer: Receives raw pixel data from the neuroimaging input; Convolutional Layers: These layers utilize filters to extract specific features from the image, focusing on various imaging characteristics and spatial orientations; Pooling Layers: Aimed at reducing the spatial dimensions (width and height) of the input data for subsequent convolutional layers. This process decreases computational demands and memory usage and enhances the network’s ability to locate features; Flattened Layer: Transforms the 2D feature matrices into a 1D vector, making it compatible with the fully connected layers that follow; Fully Connected Layers: These layers, typically dense, use the features refined and downscaled by the convolutional and pooling layers to perform classification. Each neuron in these layers connects to all activations in the preceding layer; Output Layer: Generally contains neurons equal in number to the classes being predicted. It employs a softmax activation function to generate a probability distribution across the classes; Categories/Classes: Signify the network’s final classification, denoting the category or class to which the input image is most likely to belong.

Deep Learning (DL) is a specialized subset of Machine Learning focused on processing complex input data through computer algorithms to discern meaningful features, identify underlying patterns, and make predictions (Figure 2). DL models are adept at automatically extracting relevant features from training data, thus bypassing the need for manually developing features for pattern recognition [19]. These models are structured as Artificial Neural Networks (ANNs) to mirror the neural connections in the human brain. ANNs consist of multiple interconnected layers of computational units termed nodes or “neurons”. These layers include an input layer for receiving data, several hidden layers that process and transform data to recognize patterns, and an output layer for making predictions. The connections between neurons, represented by weights, determine the strength or weakness of the message sent from one neuron to the next, thereby influencing each neuron’s output or response. Additionally, activation functions, drawing inspiration from the brain’s neural activations, are instrumental in processing the inputs received by neurons. They convert linear inputs into non-linear outputs, a critical transformation that enables the neural network to discern complex patterns. This non-linear processing facilitates the network’s capability to interpret intricate data structures and relationships, akin to solving multifaceted problems rather than performing straightforward calculations.

The training of a neural network involves adjusting its internal weights and biases to enhance the prediction accuracy. During this process, the network calculates gradients, which reflect the error change resulting from each adjustment. The error is quantified by a loss function, indicating the discrepancy between the model’s predictions and the actual outcomes. Using backpropagation, the network retroactively computes each neuron layer’s contribution to the final output error. This process is repeated across all training data samples (iterations). The weights and biases that minimize error are then determined using an optimization method known as gradient descent [19].

In Fully Connected Neural Networks (FCNNs), all neurons in adjacent layers are interconnected, forming loops. In contrast, Feed-Forward Neural Networks (FNNs) have connections only between adjacent neurons in the same layer, speeding up the learning process. Each layer’s output in an FNN is transformed by an activation function before being input into the next layer. Convolutional Neural Networks (CNNs), a type of FNN, are tailored for processing multidimensional array data, making them ideal for image analysis and spatial pattern recognition. CNNs employ small weight arrays called filters to detect specific features in a larger two-dimensional array, such as image pixels or EEG and EOG channel variations. The process of convolution transforms an image into a feature map, highlighting identical features across different image parts. These feature maps are then processed through pooling layers that consolidate similar features, and through successive convolution and pooling layers, the network recognizes more intricate patterns, like sleep stage indicators in EEG data.

Recurrent Neural Networks (RNNs) are a variant of ANNs that loop their outputs back into the network, combined with prior or future inputs. This design makes RNNs suitable for analyzing sequential time-series data, such as sleep study epochs. The integration of Long Short-Term Memory (LSTM) cells in RNNs enables them to discard irrelevant data and retain pertinent information, thus recognizing long-term dependencies in extended data segments, such as a sequence of sleep study epochs [20].

### 1.3. Sleep Staging and Cortical Arousals

There are five stages of sleep: Wake (W), Stage 1 (N1), Stage 2 (N2), Stage 3 (N3), also known as slow-wave sleep, and Rapid Eye Movement (REM) [21]. Stages 1–3 are collectively referred to as non-REM (NREM) sleep. An overnight PSG is necessary to score sleep stages. While this staging provides information about the overall structure of sleep, it does not capture its microstructure. Microarousals and cyclic alternating patterns (CAPs), for instance, are periodic electrographic activities that occur during sleep and serve as markers of increased sleep disruption and instability and are influenced by various sleep disorders such as OSA, insomnia, and PLMS. Both microarousals and CAPs are diminished with CPAP treatment for OSA [22]. As a result, they can be valuable biomarkers for diagnosing and monitoring treatment in sleep disorders that are not easily identified through standard sleep scoring. However, the manual scoring and annotation of microarousals and CAP are prone to inaccuracies, necessitating the development of automated detection methods.

An additional benefit of automated scoring programs is their potential to replace or complement the laborious scoring hours typically performed by sleep technologists, enabling them to redirect their time toward direct patient care. In 2023, the AASM initiated a two-year pilot certification program for auto-scoring software designed to classify adult sleep stages. The purpose of this program was to independently evaluate the performances of auto-scoring systems. Software companies can attain certification from the AASM, a reputable institution known for establishing standards in the field of sleep medicine.

Visual inspection is the current standard for the detection of cortical arousals on laboratory PSGs. They are clinically relevant as excessive amounts of arousals, typically due to primary sleep disorder such as OSA, cause disruption of the sleep architecture and lead to daytime hypersomnia or cognitive dysfunction. Microarousals, however, are challenging to detect with the human eye. AI can aid in the detection of these hidden abnormalities in the sleep architecture.

### 1.4. Sleep Disorders

Obstructive Sleep Apnea (OSA) occurs when the pharyngeal and retro-lingual airway collapses during sleep, leading to reduced airflow, nocturnal hypoxia, and fragmented sleep. The diagnosis of OSA relies on the apnea-hypopnea index (AHI), which measures the number of abnormal respiratory events per hour. However, the AHI alone may not accurately reflect the clinical severity of the condition. AHI values below 5 are considered normal by criteria, but they fail to capture subpopulations that exhibit more subtle symptoms and signs of sleep-disordered breathing, such as primary snoring, upper airway resistance, unrefreshing sleep, or co-existing vascular disease. There may be different subtypes of OSA that have yet to be fully understood, as the current AHI cutoffs do not adequately reflect the clinical severity or potential risks if left untreated [23].

The 12-lead Electrocardiogram (EKG) is a widely used procedure, with >100 million conducted annually in medical offices and emergency departments across the United States [24]. Leveraging the ease and speed of EKGs, they can serve to streamline the identification of at-risk patients and determine those who may require sleep studies or clinic referrals for sleep apnea. In comparison to laboratory PSG recordings, which necessitate multiple electrode placements and are therefore impractical for routine screening, EKG signals can be obtained in an office setting or potentially even in the comfort of one’s home, making routine data acquisition more feasible.

REM Sleep Behavior Disorder (RBD) is characterized by dream-enactment behavior due to impaired motor inhibition during REM sleep, a phenomenon that is referred to as REM sleep without atonia (RSWA). A confirmation for the diagnosis of RBD requires laboratory PSG data and relevant clinical symptoms. However, the interpretation of polysomnographic sleep epochs relies on subjective visual inspection, which presents a challenge for accurate interpretation. There is a need for a more reliable methodology, especially for the early detection of abnormal REM tonicity on polysomnographic data. Ongoing research is focused on early detection as it has the potential for the prevention of RBD-associated risks such as the development of alpha-synucleinopathies like Parkinson’s disease or Lewy body dementia [25].

Narcolepsy is characterized by excessive daytime hypersomnia but can be accompanied by symptoms like cataplexy (loss of muscle tone), sleep paralysis, and hypnagogic hallucinations. Diagnosing narcolepsy presents a unique challenge because excessive daytime sleepiness, its core feature, is also common in other sleep disorders, making it difficult to rely solely on this symptom for an accurate diagnosis. Narcolepsy type 1 (narcolepsy with cataplexy) is characterized by low or absent cerebrospinal fluid (CSF) hypocretin levels, which serves as a reliable biomarker [26]. The diagnosis of narcolepsy typically involves a nighttime PSG followed by multiple daytime nap opportunities through the Multiple Sleep Latency Test (MSLT), but this process is laborious and challenging to adhere to for both patients and healthcare providers. Additionally, central disorders of hypersomnia, including narcolepsy type 2, have poor retest reliability with the MSLT [27].

Periodic Limb Movements of Sleep (PLMS) are periodic leg jerks that involve the extension of the big toe, dorsiflexion of the ankle, and flexion of the knee and hip. These movements can cause microarousals and disruption of sleep continuity therefore leading to daytime hypersomnia, and potentially contribute to long-term vascular disease risk [28]. PLMS confirmation requires laboratory PSG and relies on manual identification by a sleep technician. It is also prone to inter-scorer variability.

## 2. Methods

### 2.1. Search Strategy

We conducted a search of PubMed and IEEE databases for articles published in English up until 17 September 2023. We searched PubMed using the query of MeSH terms “Sleep” AND “Artificial Intelligence” in the advanced search. We searched IEEE using the query (“All Metadata”:sleep) AND (“All Metadata”:artificial intelligence). The following systematic review was conducted by the Preferred Reporting Items for Systematic Reviews and Meta-Analyses (PRISMA) standards and registered under the ID reviewregistry1746 on Research Registry.

### 2.2. Selection Criteria

We first screened the search results against the title and abstract. We selected observational studies that reported novel artificial intelligence-based models for applications in sleep medicine. We also selected studies that externally validated previously reported models. We then screened the full-text articles of the selected studies. For a study to be included in the review, the reported model was required to meet the following criteria:

- Trained and validated with data from gold-standard diagnostic modalities for sleep staging or the diagnosis of sleep disorders. Standard diagnostics include PSG-based sleep staging, the detection of OSA using single-lead EKG signals, and a PSG-based detection of sleep disorders using AASM diagnostic criteria.
- Developed using separate training, validation, and testing datasets.
- Internally or externally validated on clinical datasets.

We excluded studies that reported models using non-gold standard diagnostic modalities such as single-channel EEGs or wearable consumer sleep technologies. We also excluded studies that reported models developed without a separate validation dataset (e.g., using cross-validation). The same criteria were used for title and abstract screening as well as for the screening of full-text articles.

### 2.3. Screening

We screened the full text of the studies selected after title and abstract screening. We broadly classified the studies that were selected for the review into two groups based on the application of the reported model. The first group included studies that reported models designed for automated sleep staging and cortical arousals. The second group included studies that reported models designed for the detection of sleep disorders.

### 2.4. Data Extraction

The following data were extracted from the selected studies and tabulated in a Microsoft Excel spreadsheet:

1. Article information including the name of the first author and year of publication.
2. Application of the reported AI model, based on which the studies were grouped into two groups (see Section 2.3).
3. Specifics of the reported AI model, including model architecture, classification tasks performed by the model, and features used for classifying the input data.
4. Composition of the training, validation, and testing datasets, including the proportion of sleep studies used in each of the three datasets, characteristics of the included patients, and the sleep study setup.
5. Performance metrics of the model on the testing dataset, including the following:
  - Agreement of the model with consensus manual scoring (measured using Cohen’s kappa), and/or accuracy of the model, and/or the F1 score for automated sleep staging models.
  - Sensitivity, specificity, and/or other reported metrics for sleep disorder detection models.
6. The full-text articles were reviewed, and relevant details about model design, population, and model performance were extracted.

The performances of different automated sleep staging models were compared based on the reported Cohen’s kappa for each model. Additionally, we compared the population studied, number of sleep study recordings, and PSG setup used in public sleep datasets that were used to develop AI models in the noted studies.

## 3. Results

### 3.1. Number of Screened and Selected Studies

**Figure 3:**
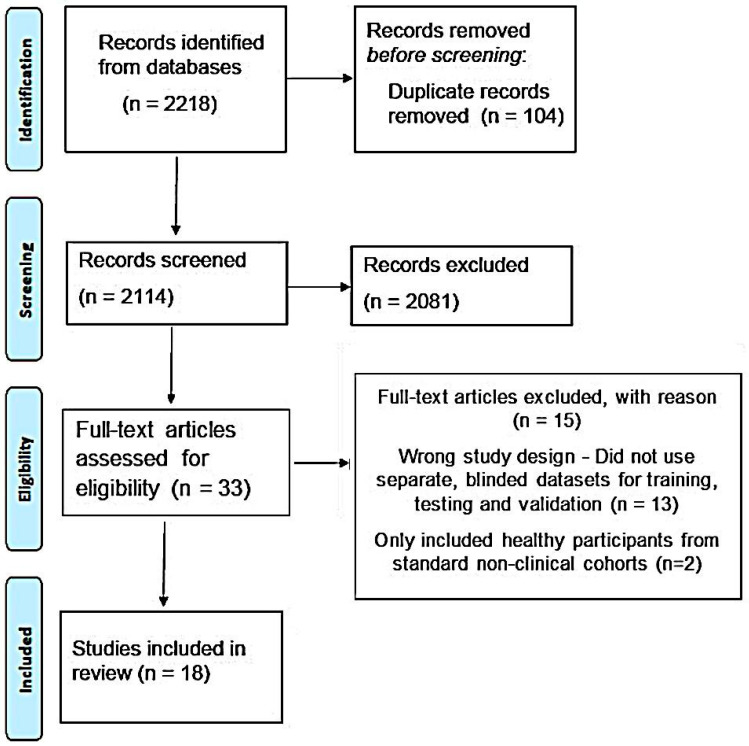
PRISMA flow diagram of the selection process for studies included in the review.

Our initial search yielded a total of 2218 results. As shown in Figure 3, 2114 results were screened against the title and abstract (after removing duplicate results) and, ultimately, 33 articles were selected for full-text screening. Of those, only eighteen studies, which met all the inclusion criteria, were included in the final analysis [29,30,31,32,33,34,35,36,37,38,39,40,41,42,43,44,45,46]. All eighteen articles reported deep learning-based models. Seventeen different models were reported in the eighteen studies that we selected [30,31,32,33,34,35,36,37,38,39,40,41,42,43,44,45,46]. One study externally validated a previously reported model [29].

### 3.2. Datasets Used for Model Development

Sixteen studies used publicly available online sleep datasets for model training, validation, and/or testing, out of which thirteen used multiple datasets from different clinical settings. Only two studies used data exclusively from patients who underwent sleep studies at the hospital where the research was conducted [30,31]. Commonly used public datasets in the included studies are compared in Table 2.

### 3.3. Automated Sleep Staging and Sleep Disorder Detection Models

Eleven of the included studies reported novel DL techniques for automated sleep staging using PSGs. Nine studies reported novel DL techniques for the automated detection of cortical arousals and detection of sleep disorders including OSA, narcolepsy, and PLMS. Two studies overlapped between both groups—Stephansen et al. reported a CNN that performed sleep staging for narcolepsy detection [32], while Biswal et al. reported a Deep Neural Network (DNN) for sleep staging, OSA screening, and limb movement detection [33].

### 3.4. Deep Learning for Sleep Staging and Cortical Arousals

#### 3.4.1. Sleep Staging

The characteristics of included studies that developed and validated automated sleep staging models are listed in Table 3, whereas their performances are listed in Table 4. In ten out of the eleven studies we selected, agreement of the model with consensus manual scoring was reported using Cohen’s kappa value. L-SeqSleepNet had the highest reported Qq/Cohen’s kappa (0.838) [34]. L-SeqSleepNet is a hierarchical RNN designed to perform long-sequence modeling, i.e., the staging of longer sequences of 200 epochs at a time. However, since the training, validation, and testing datasets were all from the SHHS database, the possibility of model overfitting must be considered. Among the models tested on external data, the Philips Somnolyzer software (Version 4.0.0) had the highest reported Cohen’s kappa of 0.78 ± 0.01 [35]. Somnolyzer 24 × 7 was first reported in 2005 for automated scoring [51]. A bi-directional LSTM version of the Somnolyzer was trained to use probability-based auto staging to generate a hypnodensity graph. Hypnodensity is a representation of the sleep stage probability distribution for each epoch. The model was tested on datasets from three separate sleep clinics (manually scored by 6 to 12 scorers). It outperformed all manual scorers [35].

#### 3.4.2. Cortical Arousals

Three out of the total eighteen studies included in the review reported cortical arousal detection models with all three being LSTM-based [36,37,38] (Table 4). Among the three models, the best performance was achieved by the bi-directional LSTM, reported by Brink-Kjaer et al., with an Area Under the Precision Recall Curve (AUPRC) of 0.82. It outperformed five out of nine expert scorers [36].

### 3.5. Deep Learning for Detection of Sleep Disorders

In Table 5, we list the characteristics of studies that developed and validated neural network models to detect sleep disorders as well as those that detect cortical arousals. Model performances are reported in Table 6. Three out of the eight studies externally validated the model on a test dataset that was different from the dataset used during training. The other five studies internally validated the model by being tested on a portion of the dataset that was not used during training.

#### 3.5.1. Obstructive Sleep Apnea

Iwasaki et al. validated their previously reported LSTM-based neural network for sleep apnea screening using EKG signals [45]. The model classifies each RR-interval from the EKG recording of a sleep study as apneic or normal, compared against RR-intervals manually annotated by PSG technologists. The model stores the label for each RR-interval from an overnight sleep study in its memory using the LSTM feature. The Apnea–Sleep (AS) ratio for the entire recording, defined as the ratio of total apnea to total sleep time, is then automatically calculated and reported. The optimal threshold to classify moderate (AHI ≥ 15 to <30) and severe (AHI ≥ 30) sleep apnea was calculated for the training dataset from a sleep clinic in Japan. The model achieved a sensitivity of 92% when tested on a different dataset from the Shiga University of Medical Science (SUMS) sleep lab in Japan. The model was also tested on 35 PSGs recorded in the 1990s from the Germany-based PhysioNet Apnea-ECG dataset, achieving a sensitivity of 95%. However, the optimal AS ratio had to first be determined using 34 PSGs from the PhysioNet dataset for training. The study datasets included patients with Chronic Obstructive Pulmonary Disease (COPD), asthma, cardiac arrhythmias, diabetes, and other potentially confounding comorbid conditions. Subjects with arrhythmias were reported to account for a significant proportion of false negative results.

Clinical predictive models for OSA are useful tools for screening at-risk patients. Kuan et al. reported a Feed-Forward Neural Network with Multilayer Perceptron (MLP) backpropagation that was trained to identify patterns suggestive of OSA based on three clinical features—age, sex and Body Mass Index (BMI)—among adult participants who had undergone full-night PSGs [31]. The three parameters predicted moderate-to-severe OSA (AHI ≥ 15) with a sensitivity of 87.7% compared against expert-scored PSG diagnoses of OSA.

#### 3.5.2. REM Sleep Behavior Disorder

Wallis et al. reported a deep 1D-CNN that was trained to detect phasic bursts and tonic activity on chin and leg electromyography (EMG) signals that were rectified to an amplitude function [30]. The model classified episodes of RSWA in accordance with AASM criteria, achieving a balanced accuracy of 91% and Cohen’s kappa of 0.68. The residual connections CNN outperformed other deep learning models that were designed and tested in the same study, including an LSTM-RNN and other CNNs with different designs.

#### 3.5.3. Narcolepsy

Stephansen et al. trained and validated an ensemble CNN for the detection of narcolepsy type I [32]. The model first performed sleep staging by generating a hypnodensity graph of sleep stage probabilities for each epoch. The model was trained to detect typical polysomnographic features of narcolepsy, including sleep latency, REM latency, and sleep onset REM periods (SOREMPs). The model also automatically extracted novel hypnodensity-based features for narcolepsy detection, ultimately creating a narcolepsy biomarker consisting of up to 38 PSG features. Finally, 90% sensitivity and 92% specificity were reported in a high-pretest probability cohort (consisting of patients who had a positive MSLT) of the testing data. This cohort, whose HLA-genotyping results were not revealed to the model, indicated that the model could differentiate Type 1 narcolepsy from other hypersomnias.

#### 3.5.4. Periodic Limb Movements of Sleep

A 2019 study from Denmark trained and validated an LSTM model that learns and extracts limb movement features from anterior tibialis EMG signals using 800 PSGs from three cohorts [46]. The memory feature enabled the model to detect PLMS, which are defined as four or more consecutive limb movements within a specified intermovement interval of 5 to 90 s. The performance of the system was compared (validated) against expert technicians and existing PLMS detectors. The LSTM achieved a maximum F1 score of 0.770 ± 0.049 for limb movement detection and 0.757 ± 0.050 for PLMS detection, performed significantly better than two human scorers, with no mean difference in F1 scores with the remaining seven scorers, and it outperformed other automatic detectors that were previously evaluated on a subset of the Wisconsin Sleep Cohort (WSC).

Biswal et al. reported a RCNN designed to extract features from tibialis anterior EMG signals to detect limb movements, providing a binary output with both periodic and isolated movements classified as limb movements [33]. A correlation of 0.7 was reported between the model-predicted and expert-scored Limb Movement Index (LMI), defined as the number of limb movements per hour.

## 4. Discussion

Our review highlights a notable gap in the availability of clinically translatable and validated ML models for integration into sleep medicine. Focusing on models that automate standard diagnostic methods and validated with independent datasets, we found that less than 1% of the reviewed studies met these criteria.

The integration of AI into sleep clinics, especially in interpreting polysomnographic data, brings forth several challenges and pitfalls that need to be addressed [52,53]. There is a pressing need for robust and generalizable ML models that are safe for clinical application. Overfitted models, which perform well on training data but poorly on new data, could pose significant risks in a clinical context. Equally concerning are underfitted models, which fail to capture the complexity of clinical data, leading to inaccurate or overly simplistic analyses. These models necessitate skilled researchers for development and implementation, therefore ensuring their generalizability in clinical cohorts and equipping healthcare providers with the necessary skills for their use. Implementing stringent independent validation and testing can prevent overfitting, a concern particularly pertinent in complex DL models that utilize numerous features [17,18]. This is especially relevant in the field of sleep medicine, as all 18 of the studies included in this review, and 30 out of the 33 articles assessed for eligibility, reported DL-based models. It is also worth noting that 13 of the included studies used clinical cohorts from different datasets for model development. External validation across diverse clinical settings is crucial to ensure the safety and adaptability of ML models in various environments that have patient demographics and different protocols, such as PSG setups and scoring rules. Researchers can potentially leverage data from several publicly available online, sleep study datasets worldwide to build models that are more universally applicable.

Our study highlights the existing research in AI applications within sleep medicine across numerous clinical and bioengineering centers. However, the field faces several challenges. Our search did not find any reported AI models for detecting a broad spectrum of common sleep disorders like insomnia, central sleep apnea, restless leg syndrome, and circadian rhythm sleep disorders that met our inclusion criteria. The cautious use of consumer wearables, not yet approved for standard medical diagnostics, must be kept in mind. Moreover, the cost of AI implementation in clinical practice and the lack of standardized PSG data across sleep laboratories, each with its specific equipment and data storage methods, pose additional challenges. Importantly, issues of data integrity, security, and ethics must be meticulously addressed, as emphasized by the AASM [54]. In the current era, AI in sleep medicine is not meant to replace clinicians but to enhance their decision-making and patient care capabilities. The AASM’s AI task force outlines five key areas for the application of big data in sleep medicine: improved diagnostic classification and accuracy; predictive treatment models; subtyping sleep disorders; the automation of sleep scoring; and patient-centered approaches to improve treatment compliance, such as with Positive Airway Pressure (PAP) therapy [55]. Ensuring the responsible use of AI and big data, addressing underfitting or overfitting, and maintaining patient privacy are imperative in advancing these goals.

It is important to note the limitations of our review. Due to time constraints and the broad scope of our query, we could not search databases such as Scopus. Hence, our search may not have retrieved all the pertinent literature. However, we conducted a search of both a clinical (PubMed) and an engineering-related (IEEE) database.

## 5. Conclusions

There remains a critical need for ML models to undergo thorough validation and established reliability before they can be broadly implemented in clinical sleep medicine. The scarcity of studies reporting independent validation datasets in the intersection of sleep medicine and AI underscores the importance of rigorous validation and testing to substantiate the efficacy of new AI models. This necessitates a concerted effort for more comprehensive studies that focus on validating ML models across diverse and heterogeneous populations drawn from clinical cohorts.

## Figures and Tables

**Table 1:** Essential concepts and analogies in Machine Learning.

Term	Definition	Example
Iterations	Repetitive cycles where an ML model practices making predictions using training data.	Like a basketball player practicing free throws multiple times to improve.
Features	Traits or details in the data that help in categorizing or analyzing the data.	In a smartphone camera’s photo-sorting app, features might include color, brightness, or the presence of faces to categorize images into different albums.
Cross-validation	It is technique in statistical analysis and Machine Learning with which a dataset is divided into multiple parts. These parts are then used interchangeably as training and testing sets to validate the accuracy and generalizability of a model. This process helps in assessing how well a model will perform on an independent dataset and in preventing overfitting.	Like a coach dividing the team into several groups and having each group play both roles of the main team and the opponent in different matches. This helps the coach understand how well the team adapts to different scenarios. However, this does not guarantee that the team will perform equally well against actual external opponents
Overfitting	When an ML model learns the training data too well but struggles with new data.	A student who memorizes answers for a specific set of questions for a history test cannot apply the knowledge to new, unseen questions on the same topic.
Underfitting	This occurs when an ML model is not complex enough to capture the underlying patterns and relationships in the training data, leading to poor performances on both training and new, unseen data.	Like trying to understand a complex novel by only reading the summary, and as a result, being unable to grasp the full story or discuss it in detail.
Weight	The importance given to a message passed between neurons in a neural network.	Similar to adjusting the balance on a music mixer to control how much each instrument contributes to the overall sound of a band.
Biases	In a neural network, “bias” is an adjustable parameter that enables the model to modify its output independently of the input data, playing a crucial role in determining neuron activation and thus influencing the model’s overall behavior and accuracy.	In a job application screening system trained mostly on resumes from a few top universities, there may be a bias favoring applicants from those schools, potentially overlooking equally qualified candidates from other institutions.
Activation Function	An activation function in a neural network is a mathematical formula that determines whether and to what extent a neuron should be activated, based on the input it receives. It helps the network make non-linear decisions, allowing it to handle complex data patterns.	In a photo-filtering app, an activation function might decide how strongly a certain feature, like brightness or color saturation, should influence whether a photo is categorized as “outdoor” or “indoor”.
Loss Function	How much the model’s prediction differs from the actual result.	The difference between a GPS’s estimated time of arrival and the actual time you reach your destination.
Gradient	The gradient refers to the measure of change in the network’s error (or loss) in response to adjustments in its weights and biases.	How changing the amount of sugar in a cake recipe affects its sweetness when the goal is to find the ideal sweetness (accuracy) of the product.
Backpropagation	A process that calculates how each part of the network contributed to the error.	Analyzing which step in a baking recipe went wrong when the cake does not rise.
Gradient Descent	A method to find the best weights and biases for the lowest error in a network.	Searching for the perfect oven temperature and baking time for the ideal cake texture.

**Table 2:** Characteristics of commonly used public sleep datasets.

Dataset	Characteristics of Included Subjects	Number of Recordings	PSG Setup
Massachusetts General Hospital (MGH)-PSG [33]	Symptomatic	10,000 (10,000 subjects)	Six-channel EEG, EOG, chin and leg EMG, EKG, SaO_2_, chest and abdomen movement sensors, nasal airflow, pressure transducer (PTAF), position
Sleep Heart Health Study (SHHS) [47]	Adults over 40 with and without a history of snoring who were previously recruited to epidemiological cohort studies on cardiovascular health	9376 (5804 subjects)	Two-channel EEG, EOG, EKG, SpO_2_, chest and abdomen movement sensors, nasal airflow
Sleep EDF (expanded) [48]	Symptomatic (difficulty falling asleep) subjects and healthy controls	197 (96 subjects)	Two-channel EEG, EOG, chin EMG, SaO_2_, chest and abdomen movement sensors, nasal airflow
Stanford Sleep Cohort (SSC) [49]	Symptomatic	760 (760 subjects)	Six-channel EEG, EOG, chin and leg EMG, EKG, SaO_2_, chest and abdomen movement sensors, nasal airflow
Wisconsin Sleep Cohort (WSC) [11]	Combination of subjects at high and low risk for OSA based on questionnaire responses	2570 (1549 subjects at baseline, ongoing follow-up)	Two-channel EEG (six channels in select recordings), EOG, chin and leg EMG, EKG, SaO_2_, chest and abdomen movement sensors, nasal and oral airflow, nasal pressure, position
Institute of Systems and Robotics—University of Coimbra (ISRUC) [50]	Symptomatic patients with diagnosed sleep disorders and healthy controls	126 (118 subjects)	Six-channel EEG, EOG, chin and leg EMG, SaO_2_, chest movement sensor, nasal airflow

EEG—Electroencephalogram; EOG—Electrooculogram; EMG—Electromyogram; EKG—Electrocardiogram; SaO_2_—Oxygen saturation of arterial blood; SpO_2_—Oxygen saturation of peripheral blood; EDF—European Data Format.

**Table 3:** Characteristics of studies that developed and validated automated sleep staging models.

First Author, Year	Type of Neural Network	Training and Validation Datasets	Testing Datasets	Comments
Patanaik, 2018 [39]	Deep CNN	1046 PSGs of healthy adolescents (DS1) * 284 PSGs of healthy adults (DS2) 75% for training and 25% for validation	210 PSGs of adolescent and adult patients with suspected sleep disorders (DS3) 77 PSGs of Parkinson’s disease patients, 42% of whom were classified as having REM sleep behavior disorder (RBD) and 28% as probably having RBD (DS4)	Trained on data from healthy subjects, tested on data from patients with suspected sleep disorders and RBD
Biswal, 2018 [33]	RCNN	9000 training and validation PSGs from the MGH dataset 5224 from the SHHS dataset	1000 held out PSGs from MGH 580 held out PSGs from SHSS	
Stephansen, 2018 [32]	Ensemble of Cross-Correlation (CC) encoded CNN models (Stanford STAGES model)	3507 (90% training, 10% validation) from the WSC, SSC, and KHC datasets	70 PSGs scored manually by six scorers from the IS-RC cohort (staging)	
Olesen, 2020 [40]	CNN (feature extraction) + RNN (staging)	15,684 PSGs from ISRUC, MrOS, SHHS, SSC, and WSC datasets (87.5% training, 2.5% validation, 10% testing)	10% PSGs held out from the same datasets	Same accuracy obtained using 75% training data (four out of five cohorts) as 100% (five out of five cohorts)
Abou Jaoude, 2020 [41]	Multi-modal DNN	5041 PSGs for training 650 for validation From the MGH-PSG dataset	HomePAP—243 PSGs ABC—129 PSGs MGH-PSG—650 PSGs	Developed a PSG-based staging model (CRNN-PSG) and fine-tuned it into a scalp EEG-based staging model (CRNN-EEG); only the performance of the PSG-based model is considered
Zhang, 2020 [42]	LSTM-RNN	122 training and 20 validation PSGs of adults with a history of snoring performed at Beijing Tongren Hospital	152 PSGs from the study dataset 40 PSGs from 20 SC subjects from the SleepEDF dataset	Compared performance of staging model between participants with and without OSA
Alvarez-Estevez, 2021 [43]	CNN	354 PSGs (80% of a total 443) from six datasets further split into 80% for training and 20% for validation	89 PSGs (20% of a total 443) held out from the six study datasets that included ISRUC and SHHS	
Cesari, 2021 [29]	Ensemble of (CC) encoded CNN models (Stanford STAGES model)	1066 PSGs from the Study of Health in Pomerania-TREND	Externally validated, previously developed Stanford STAGES model (see above)	
Guillot, 2021 [44]	RNN	5788 total PSGs from eight datasets including the MASS, SleepEDF, MrOS, and SHHS datasets Did not specify the split between the training, validation, and testing sets	Testing dataset was unseen during training in a Direct Transfer (DT) setting Both of the other settings required the use of cross-validation (CV); their performance was not considered for the review	Designed to classify sequences of multiple epochs at once PSG settings varied across datasets and included 2, 3, 5, 8, and 12 lead EEGs with or without EMG.
Bakker, 2023 [35]	Bi-directional LSTM-RNN (Somnolyzer)	588 PSGs from SIESTA dataset	95 PSGs from three clinical datasets separate from the training set; datasets A (*n* = 70), B (*n* = 15), and C (*n* = 10) were scored by 6, 9, and 12 scorers, respectively.	
Phan, 2023 [34]	Hierarchical RNN	3824 (70% of 5463) PSGs from SHHS dataset with 100 PSGs held out for validation	1639 (30% of 5463) PSGs from the SHHS dataset	

* Cognitive Neuroscience Lab, Duke-NUS Medical School, Singapore (DS1); Chronobiology and Sleep Lab, Duke-NUS Medical School, Singapore (DS2); Sleep Disorders Unit, Singapore General Hospital, Singapore (DS3); Laboratory for Sleep and Chronobiology, University of California San Diego, School of Medicine, USA (DS4). Abbreviations: PSG—Polysomnogram; SHHS—Sleep Heart Health Study; MGH—Massachusetts General Hospital; WSC—Wisconsin Sleep Cohort; SSC—Stanford Sleep Cohort; KHC—Korean Hypersomnia Cohort; IS-RC: Inter-Scorer Reliability Cohort; EDF—European Data Format; ISRUC—Institute of Systems and Robotics, University of Coimbra Sleep Cohort; HomePAP—Home Positive Airway Pressure; ABC—Apnea, Bariatric surgery, and CPAP; RBD—REM Sleep Behavior Disorder; OSA—Obstructive Sleep Apnea; CNN—Convolutional Neural Network; RNN—Recurrent Neural Network; RCNN—Region-based Convolutional Neural Network; LSTM—Long Short-Term Memory.

**Table 4:** Performances of automated sleep staging models.

First Author, Year	Type of Neural Network	Model Performance on Testing Dataset		
		Cohen’s Kappa	Accuracy	F1 Score
Patanaik, 2018 [39]	Deep CNN	0.740 (DS3); 0.597 (DS4)	81.4% (DS3); 72.1% (DS4)	
Biswal, 2018 [33]	RCNN	80.5 (MGH); 73.2 (SHSS)	87.5% (MGH); 77.7% (SHSS)	
Stephansen, 2018 [32]	CNN	57.7 ± 6.1 (Unbiased overall Cohen’s kappa across six scorers)	86.8 ± 4.3	
Olesen, 2020 [40]	CNN + RNN	0.728, 95%CI: 0.726–0.731 (Mean of all combinations of four training and one validation cohorts)		
Abou Jaoude, 2020 [41]	Multi-modal DNN	0.64 (For CRNN-PSG model on both testing datasets)		
Zhang, 2020 [42]	LSTM-RNN	0.7276 (study dataset) 0.77 for (SleepEDF)	0.8181 (study dataset); 0.836 (SleepEDF)	0.8150 (study dataset); 0.781 (SleepEDF)
Alvarez-Estevez, 2021 [43]	CNN	0.63 (Average kappa on external datasets)		
Cesari, 2021 [29]	CNN	0.61 ± 0.14 (Overall manual vs. auto scoring for both datasets)		
Guillot, 2021 [44]	RNN			64.9 to 84.4 across eight datasets 84.4 for DOD-H dataset (12-lead EEG)
Bakker, 2023 [35]	LSTM-RNN	0.78 ± 0.01 compared to unbiased consensus of scorers for auto-scoring model (vs. 0.69 ± 0.063 for best manual scorer)		
Phan, 2023 [34]	RNN	0.838 (SHHS)		

Abbreviations: PSG—Polysomnogram; SHHS—Sleep Heart Health Study; MGH—Massachusetts General Hospital; DOD-H—Dreem Open Dataset-Healthy; EDF—European Data Format; CNN—Convolutional Neural Network; RNN—Recurrent Neural Network; RCNN—Region-based Convolutional Neural Network; LSTM—Long Short-Term Memory.

**Table 5:** Characteristics of studies that developed and validated models for the automated detection of sleep disorders and cortical arousals.

First Author, Year	Type of Neural Network	Disease Classified (Present vs. Absent)	Features	Training and Validation Datasets	Testing Dataset
Stephansen, 2018 [32]	Ensemble of Cross-Correlation (CC) encoded CNN models (used as biomarker of narcolepsy)	Narcolepsy Type 1	Hypnodensity of sleep stages, sleep latency, REM latency, SOREMPs *, etc.	645 training, 445 validation PSGs from seven cohorts: WSC, SSC, KHC, AHC, JCTS, IHC, and DHC	321 PSGs from two cohorts never seen by the model: FHC and CNC
Iwasaki, 2022 [45]	LSTM-RNN	Severe OSA (AHI ≥ 30); Moderate-to-severe OSA (AHI ≥ 15)	Ratio of total apnea to total sleep duration (AS ratio) based on RR intervals labeled as apneic or normal	938 adolescent (>12 yrs) and adult PSGs (468 training, 470 validation) performed at the Nakamura clinic in Okinawa, Japan	SUMS dataset—N = 59 PhysioNet dataset—N = 35 (34 PSGs used to determine AS ratio threshold)
Kuan, 2022 [31]	ANN	Moderate-to-severe OSA	Age, sex, BMI	7328 manually scored full-night PSGs of adult patients who had not been previously treated for OSA	2094 held out PSGs from the same lab
Pourbabaee, 2019 [37]	Dense Recurrent CNN (DRCNN) with bi-directional LSTM	Primary classification task: non-apnea/hypopnea arousal (target arousal) vs. apnea/hypopnea vs. normal sleep vs. wake	EEG, EMG, EOG, SpO_2_	1985 PSGs from the MGH dataset: 794 training, 100 validation, 100 testing	989 held out PSGs from the same dataset
Brink-Kjaer, 2020 [36]	LSTM-RNN	Cortical arousal vs. sleep; wake vs. sleep	EEG, EOG, EMG	Training: 2889 Home Sleep Test (HST) from MrOS, CFS Validation: 996 PSGs from MrOS, CFS and in-lab PSGs from WSC	30 unseen PSGs, each from SSC (clinical) and WSC annotated by nine sleep technicians
Li, 2020 [38]	LSTM-RNN	Cortical arousal detection	Single-lead EKG signal	MESA cohort: 1112 training and 124 test PSGs SHHS cohort: 1058 training and 118 test PSGs	311 unseen PSGs from MESA and 785 from SHHS
Wallis, 2020 [30]	1D-CNN with residual connections	REM sleep with Atonia	Amplitude and sustained duration of chin and leg EMG compared to predefined baseline for short phasic (P) bursts and longer tonic (T) events; EEG and EOG for staging	554 training and 60 validation in-hospital PSGs manually scored and annotated for T and P events	78 unseen PSGs from the same dataset
Carvelli, 2020 [46]	LSTM-RNN	Limb movement score calculation	Left/right anterior tibialis (LAT/RAT) EMG signal used to calculate Periodic Limb Movements as per AASM criteria; EKG signal used to filter out artifacts in LAT/RAT signal	655 training and 53 test PSGs from the MrOS, WSC, and SSC datasets manually scored and annotated for limb movements	92 unseen PSGs from the same datasets
Biswal, 2018 [33]	RCNN	OSA detection Limb Movement Detection	Chest, abdomen movements, SaO_2_ (for AHI) LAT/RAT EMG (for limb movements)	9000 training and testing PSGs from the MGH dataset; 5224 from the SHHS dataset	1000 held out PSGs from MGH 580 held out PSGs from SHSS

* REM sleep occurring after at least 2.5 min of wake or stage 1; Abbreviations: PSG—Polysomnogram; SHHS—Sleep Heart Health Study; MGH—Massachusetts General Hospital; WSC—Wisconsin Sleep Cohort; SSC—Stanford Sleep Cohort; KHC—Korean Hypersomnia Cohort; AHC—Austrian Hypersomnia Cohort; JCTS—Jazz Clinical Trial Sample; IHC—Italian Hypersomnia Cohort; DHC—Danish Hypersomnia Cohort; FHC—French Hypersomnia Cohort; CNC—Chinese Narcolepsy Cohort; CFS—Cleveland Family Study; MESA—Multi-Ethnic Study of Atherosclerosis; SOREMPs—Sleep Onset REM Periods; RCNN—Region-based Convolutional Neural Network; LSTM—Long Short-Term Memory; RNN—Recurrent Neural Network; ANN—Artificial Neural Network; AHI—Apnea Hypopnea Index; OSA—Obstructive Sleep Apnea; SUMS—Shiga University of Medical Sciences; 1D-CNN—One-Dimensional Convolutional Neural Network.

**Table 6:** Performance of models for the automated detection of sleep disorders.

First Author, Year	Type of Neural Network	Disease Classified (Present vs. Absent)	Model Performance
Stephansen, 2018 [32]	CNN	Narcolepsy Type 1	Sensitivity = 93%; Specificity = 91%; Accuracy = 0.92; PPV = 0.87; NPV = 0.95; for narcolepsy biomarker in never-seen replication sample Sensitivity = 90%; Specificity = 92%; for narcolepsy biomarker in HPT cohort
Iwasaki, 2022 [45]	LSTM-RNN	Severe OSA (AHI ≥ 30); Moderate-to-severe OSA (AHI ≥ 15)	For the detection of moderate-to-severe OSA (AHI ≥ 15): SUMS dataset: AUC = 0.93; Sensitivity = 0.92; Specificity = 0.89; PhysioNet dataset: AUC = 0.95; Sensitivity = 0.95; Specificity = 0.86
Kuan, 2022 [31]	ANN	Moderate-to-severe OSA	For predicting moderate-to-severe OSA: Accuracy = 76.4%; Sensitivity = 87.7%; Specificity = 56.9%; PPV = 77.7%; NPV = 73.0%
Pourbabaee, 2019 [37]	Dense Recurrent CNN (DRCNN) with bi-directional LSTM	Primary classification task: non-apnea/hypopnea arousal (target arousal) vs. apnea/hypopnea vs. normal sleep vs. wake	AUROC = 0.931 AUPRC = 0.543 (For non-apnea/hypopnea arousal detection on blind test set)
Brink-Kjaer, 2020 [36]	LSTM-RNN	Cortical arousal vs. sleep; wake vs. sleep	Mean F1 score of the model for arousal detection in unseen testing dataset = 0.76 Mean precision = 0.72; Recall = 0.81; AUPRC = 0.82
Li, 2020 [38]	LSTM-RNN	Cortical arousal detection	AUPRC = 0.39 AUROC = 0.86 For model trained on MESA and validated on SHHS
Wallis, 2020 [30]	CNN	REM sleep with Atonia	Balanced accuracy (BAC) = 0.91; Cohen’s Kappa 0.68
Carvelli, 2020 [46]	LSTM-RNN	Limb movement score calculation	Maximum F1 score = 0.770 ± 0.049 for LM and 0.757 ± 0.050 for PLMS
Biswal, 2018 [33]	RCNN	OSA detection Limb movement detection	r^2^ for AHI scoring = 0.85; r^2^ for limb movement detection = 0.7 (MGH dataset only)

Abbreviations: SHHS—Sleep Heart Health Study; MGH—Massachusetts General Hospital; MESA: Multi-Ethnic Study of Atherosclerosis; PPV—Positive Predictive Value; NPV—Negative Predictive Value; HPT—High Pretest Probability Cohort; AHI—Apnea Hypopnea Index; OSA—Obstructive Sleep Apnea; SUMS—Shiga University of Medical Sciences; AUROC—Area Under Receiver Operating Curve; AUPRC—Area Under Precision Recall Curve.

## Data Availability

Data sharing is not applicable.
